# Acute bilateral loss of vision as organic manifestation of repressed psychological stress: A case report

**DOI:** 10.1002/ccr3.4809

**Published:** 2021-09-22

**Authors:** Ruzita Jamaluddin, Ammar Azhar Izham, Ahmad Fadzil Abdul Hamid, Wan Nadiah Amir Hamzah, Muhamad Ruzaini Abd Hamid

**Affiliations:** ^1^ Department of Psychiatry and Mental Health Hospital Tuanku Fauziah, Kangar, Perlis Ministry of Health Malaysia Malaysia; ^2^ Clinical Research Centre Hospital Tuanku Fauziah, Kangar, Perlis Ministry of Health Malaysia Malaysia; ^3^ Department of Ophthalmology Hospital Tuanku Fauziah, Kangar, Perlis Ministry of Health Malaysia Malaysia

**Keywords:** blindness, conversion disorder, dysthymic disorder, stress, vision

## Abstract

Comprehensive clinical assessment with integrated team approach is crucial in managing cases of non‐organic visual loss. Apart from pharmacotherapy, psychosocial rehabilitation should also be adequately addressed.

## Graphical abstract

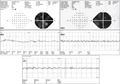

## 1 INTRODUCTION

Herein, is a rare case of dysthymic disorder complicated with visual conversion disorder, presented as painless acute bilateral loss of vision. Extensive ophthalmologic workup revealed equivocal results. However, upon referral to a psychiatrist, significant psychological stressors were identified and adequately addressed with psychotherapy and psychological rehabilitation, gradually restoring functional vision.

Non‐organic visual loss refers to visual disturbances in the absence of structural dysfunction along the pathway between the cornea and occipital cortex.^1^ This rare diagnosis constitutes to only up to 5% of patients seen by ophthalmologists, eventually determined to be either malingering, or visual conversion disorder.^2^ The latter requires in‐depth exploration into the social and emotional state of the patient to relate with the presenting symptoms.

Visual conversion disorder is a type of dissociative sensory loss disorder characterized by unilateral or bilateral visual impairment in the absence of organic cause. The patient is not consciously aware of the relationship between the repressed psychological stresses with the presented visual disturbance until detailed psychosocial history was explored by a trained psychiatrist.

Herein, we aimed to delineate the importance of a comprehensive ophthalmologic assessment in a patient presented with acute bilateral loss of vision and discuss the scientific basis of visual manifestation of repressed psychological stress.

## 2 CASE REPORT

This is a case of a 30‐year‐old gentleman with no prior medical or psychiatric illness. He presented to us with sudden onset of bilateral painless loss of vision immediately following an accidental head injury resulting from a backward fall from a 3‐meter height pickup truck. He developed cerebral concussion and sustained superficial laceration wound with minimal bleed. Ophthalmologic assessment at the emergency department within the next hour of injury was recorded as counting fingers. Otherwise, he was alert and oriented with full Glasgow‐coma scale. Other systemic examination revealed no abnormalities and neurological examination was unremarkable.

**FIGURE 1:**
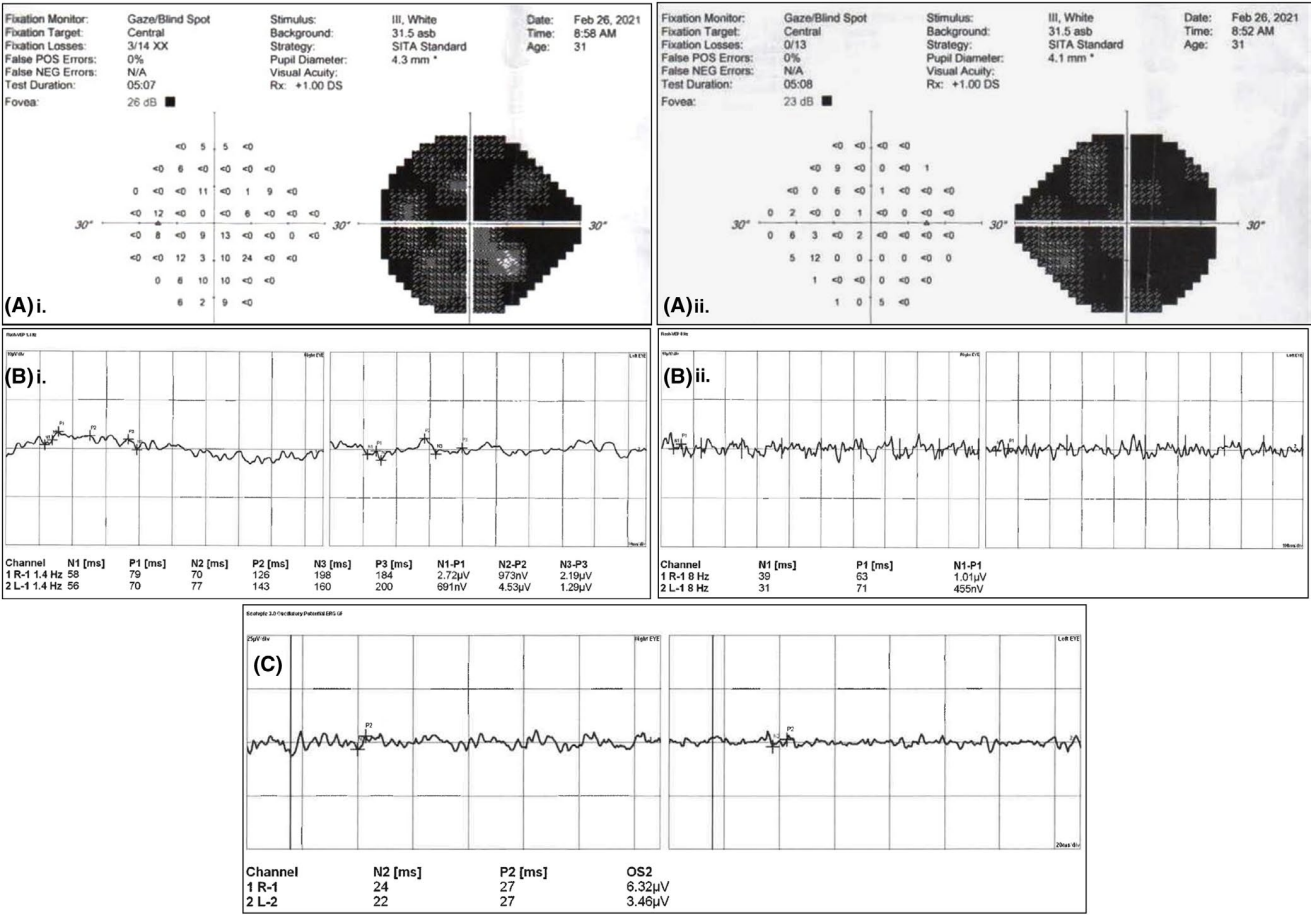
Unremarkable ophthalmologic diagnostic findings. (A) Non‐specific visual field defect on Humphrey's visual field testing; (B) Normal visual evoked potential testing; (C) Normal scotopic ERG

Detailed ocular examination the next day suggested bilateral no light perception on visual acuity testing. The differential diagnoses include cortical blindness and traumatic optic neuropathy. Hence, further tests were performed including anterior and posterior segment ocular examination, relative afferent pupillary defect (RAPD) test, fogging test, and menace reflex, which were unremarkable. Computed tomography and magnetic resonance imaging of the brain also revealed no abnormalities. Visual Evoked Potential (VEP) was normal, suggesting no abnormalities of the visual pathway and cortex, and electroretinography (ERG) indicates no pathologic process. In view of the inconclusive diagnostic findings (Figure 1), the patient was subsequently referred for a psychiatric evaluation to rule out conversion disorder.

Comprehensive psychiatric assessment identified significant enduring traumatic events and psychosocial stressors that could potentially contribute to conversion disorder. The patient came from a lower socio‐economic background with poor psychosocial family support. Being the sole breadwinner to support his wife, a 10‐month‐old daughter, and elderly parents, he took multiple odd jobs with very little time for himself. Despite the meager pay check, his lack of assertiveness and extreme generosity greatly affected his occupational and social life.

His mental status examination revealed persistent depressive symptoms with low self‐esteem, feeling of anhedonia, hopelessness, indecisiveness and worthlessness. He also complained of loss of energy, loss of appetite and insomnia. He appeared frail and weighed at only 110 lbs. His affect was restricted, and his speech was relevant and coherent with normal amount, but with low volume and rate. There was no psychomotor retardation or agitation, and his cognitive functions were intact. He was diagnosed with dysthymic disorder with concomitant visual conversion disorder. Pharmacotherapy with antidepressants, Fluoxetine 20 mg daily was gradually increased to 40 mg daily. Supportive psychotherapy, family interventions, psychoeducation and modified cognitive behavioral therapy (CBT) were prescribed.

Early phase of the therapy resulted in oral non‐compliance and patient's hesitancy toward psychotherapy in view of patient's denial of the clinical diagnosis. Proper counselling helped him develop good insight of his condition with subsequent improved treatment compliance. His first noticeable symptom recovery was noted within 6–9 months later. His visual acuity gradually improved from counting finger to 6/7.5, bilaterally. His color blindness improved from mere black‐and‐white to differentiation of color tones. However, the color blindness appeared to fluctuate and persist to a minimum.

## 3 DISCUSSION

Our patient presented with sudden onset of bilateral complete loss of vision following accidental trauma. Detailed ocular examination and brain imaging revealed no significant abnormality that could have contributed to his visual disturbance. Subsequent referral to a trained psychiatrist explored the presence of significant psychosocial stressors that may manifest as visual conversion disorder, potentially triggered by the accidental traumatic event.

Visual disturbance occurring in the absence of ocular or non‐ocular pathology accounts for approximately 1%–5% of ocular symptoms seen by ophthalmologists.^1^ Cases of malingering that convincingly mimic true blindness are rare as people lack the intuitive knowledge of visual pathways, whereas a conversion reaction presents with physical symptom to express the internal psychological conflict. Such condition is also referred to as non‐organic or functional vision loss,^3^ or somatoform disorders.^4^

A diagnosis of non‐organic visual loss is made when the visual acuity proved to be better than the subjectively alleged, and when the functional integrity of the afferent visual pathway is confirmed by clinical examination, with or without supplementary electrophysiological investigations. Other observations that may not be consistent with a specific organic etiology include a smooth entrance into the consulting room, an intact menace reflex, flinching with increased illumination, and failure to direct eyes toward their own hands during manual tasks. Additionally, spinning a vertically striped surface will elicit an optokinetic nystagmus, unless true blindness is present. Likewise, having the patient stare at a large moving mirror irresistibly compels them to follow their own image.

Apart from the various ophthalmologic tests performed with equivocal results in our patient, an electroencephalogram (EEG) that was not done, may also help. Intact visual system can be determined by the presence of alpha rhythm overlying the occipital lobes in patients at rest with their eyes closed, which will be loss when the eyes are opened.

The underlying pathophysiology of visual conversion disorder remains obscure, and the body of evidence for stress‐induced vision loss is rather limited. However, stress correlates well with circulating cortisol level, and persistent hypercortisolism is hypothesized to result in vascular dysregulation and repetitive activation of the sympathetic nervous system that reduces glandular activities, resulting in reduced lacrimation and dry eyes with impaired vision.^5^, ^6^ Additionally, psychological stress is also known to cause tension in the ocular muscles and tissues, altering the shape of the eyeball, hence affecting visual acuity.^6^

Treatment challenges in such cases include convincing patient regarding the nature of the illness. Our patient was in complete denial during the initial phase of psychiatric intervention. This could have been partially contributed by the social stigma of psychiatric illness in the community, resulting in treatment delay, hence poor recovery of symptoms. The natural history of non‐organic visual loss should be explained, and the expectation of complete visual recovery should be adequately expressed.

Additionally, individuals with certain personality patterns and maladaptive coping mechanism risk somatic problems upon exposure to prolonged stress,^3^, ^4^, ^5^ as exhibited by our patient. Stress is a vicious cycle, leading to vision loss and further worsen the stress condition. In most cases of psychological blindness, the symptoms will resolve spontaneously following psychological rehabilitation,^7^, ^8^, ^9^ and the timeline for eventual visual recovery depends on case‐to‐case basis.^10^

## 4 CONCLUSION

Our case exemplifies the importance of comprehensive assessment with integrated team approach when dealing with the challenging case of non‐organic vision loss. Apart from pharmacotherapy, the main aspect of psychological rehabilitation in visual conversion disorder includes addressing the psychosocial concerns. Continuous ophthalmologic assessment acts as the safety net to monitor patient progress until complete resolution of symptoms.

## CONFLICT OF INTEREST

None declared.

## AUTHOR CONTRIBUTIONS

RJ, AFAH, and WNAH conceptualized and designed the study, and together with AAI, were involved in data acquisition and interpretation. RJ, AAI, AFAH, and WNAH were involved in drafting of the article and MRAH revised it critically for important intellectual content. All authors were involved in the clinical management of the patient, and approved the final version of the manuscript prior to submission. RJ and MRAH agreed to be accountable for the article and to ensure that all questions regarding the accuracy or integrity were investigated and resolved.

## ETHICAL APPROVAL

This project was registered with the National Medical Research Register of the Ministry of Health Malaysia (NMRR‐21‐693‐59633). The research was performed in accordance with relevant guidelines and regulations recommended by the institution.

## CONSENT

Written informed consent for publication was obtained from the patient.

## Data Availability

Data sharing is not applicable to this article as no new data were created or analyzed in this study.
